# Generalization of biological foundation models requires prospective validation across contexts

**DOI:** 10.1016/j.patter.2026.101665

**Published:** 2026-10-09

**Authors:** Lucas A. Gillenwater, Casey S. Greene

**Affiliations:** 1 Department of Biomedical Informatics, University of Colorado Anschutz, Aurora, CO, USA

## Abstract

Biological foundation models promise generalization. The authors argue that the field needs a community-driven evaluation that tests generalization through prospective validation across biological and experimental contexts.

## Main text

We hope foundation models will learn the rules of molecular regulation. However, foundation models do not output rules. Researchers evaluate models on task performance (e.g., cell-type annotation, perturbation prediction, and batch integration). For foundation models to earn trust and have a clinical impact, evaluations must map the boundaries of generalization.^1^ Models must show predictive accuracy across labs, donors, tissues, protocols, modalities, and perturbations. Here, we propose that foundation models require prospective evaluations that specifically test generalization. We define prospective evaluations as those that rely on public, verifiable predictions before experimental results are revealed to model builders.

Recent publications show generalization gaps. Steiner et al. benchmarked single-cell foundation models (scGPT,^2^ Geneformer,^3^ and scFoundation^4^) on cell-type classification and reported that foundation models did not outperform linear baselines.^5^ Similarly, Ahlmann-Eltze et al. found that additive baselines outperformed the aforementioned single-cell models and others in predicting transcriptome changes from perturbations not observed in the training data.^6^ In both cases, outside groups included simple baselines that negated the claimed advantages of more complex models.

The Virtual Cell Challenge in 2025 sought to blind model developers from evaluation data but struggled with scoring metrics for biological inference. The organizers asked entrants to predict single-gene perturbation effects in H1 human embryonic stem cells, a context held out of training. Over 300 teams made final submissions. The organizers reported that models did not consistently beat naive baselines across all metrics (i.e., perturbation discrimination score, differential expression score, and mean absolute error). The published design states that minimum thresholds prevent optimizing for one metric at the expense of others.^7^ In practice, the organizers capped the mean absolute error score, and participants reported simple data transformations that inflated the variance and outscored top models. Independent analysis shows that the perturbation discrimination score shifts with the choice of distance measure and scale of predictions.^8^ A single held-out benchmark turns leaderboards into loss functions.

The Virtual Cell Challenge mirrors how developers present their own models. A developer picks the evaluation context, baseline, metric, and subset of predictions to show. Any experimental combination that yields weak results can be revised before publication. Groups rarely register hypotheses in advance or present negative results. Filtering predictions to a confident subset narrows the claim by narrowing the search space.

George Box argues that all models are wrong and some are useful. We do not reject that adage. A perturbation model that shrinks the experimental search space is worth running. However, this position shifts the argument to quantifying the level of shrinking through evaluation at the bench. A wet bench biologist will wonder how many of the top 50 model predictions result in a true positive. How does that compare with ranking candidates with a linear model or pathway database? Precision at k predictions on prospective experiments checks the claim for narrowing the search space in that context. Generalization measures how the score changes as the context changes.

We therefore propose that each model should report two types of scores. The *in silico* score shows performance on held-out, retrospective data. A prospective score comes from wet-lab experiments designed to test predictions the models logged in advance. The gap between the metrics is the model’s generalization gap (Figure 1A). A model that scores well *in silico* but poorly on prospective experiments has fit the training data. One gap measures one context. Mapping over multiple contexts shows the extent of generalization (Figure 1B). Experiments in precision oncology have run this comparison. Preclinical studies suggested synergy in combination therapy, yet additive models predicted clinical outcomes well.^9^ The preclinical inference was the *in silico* score, and the trial outcome was the prospective score. Differences exposed the generalization gap of synergy models.

**Figure 1:**
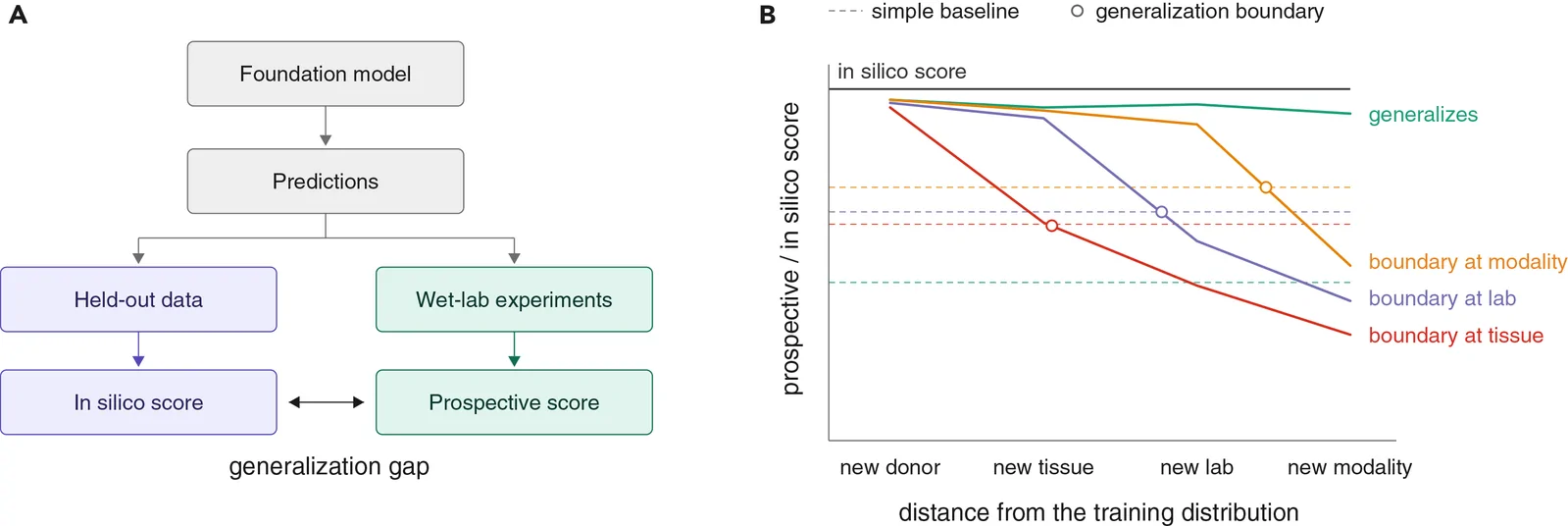
Mapping the generalization gap (A) Foundation model produces predictions. Scoring them against held-out retrospective data yields an *in silico* score. Scoring the same predictions against wet-lab experiments designed after the predictions are logged yields a prospective score. The difference between the two is the generalization gap in that context. (B) Schematic. Prospective score, normalized to the *in silico* score, across a designed sequence of tranches ordered by distance from the training distribution. Dashed lines mark the simple baseline in each scenario, which varies by context.

No single group can test every prediction. One workable version is a tiered community standard, built the way the Critical Assessment of Strucutre Prediction (CASP) was built for protein structure.^10^ A neutral party holds the experimental design, releases the inputs, collects the logged predictions in a shared evaluation harness, and then releases the measured outputs with scores. To ensure neutrality, some tranches must come from labs with no model in the evaluation. Each tranche is spent when released. Models trained after a tranche closes become new models in the next tranche.

Whether evaluation is driven by a large singular effort or distributed efforts, labs already generate data across new donors, tissues, and protocols. Converting these to an ongoing prospective evaluation framework requires that the data stay embargoed until predictions are logged. Mapping where a model fails takes far less data than training one. Designed data tranches can map the boundaries of generalization across new labs, donors, tissues, protocols, modalities, and perturbations. Each tranche should include positive controls, negative controls, and simple baselines to contextualize the scores.

We do not know which advancement in models will close the generalization gap. Until models are scored on experiments they have not seen, run by people who do not need them to succeed, we will continue to struggle to say which advance worked. The present state leaves us with many models and no test we can trust.

## Declaration of interests

The authors declare no competing interests.

## Declaration of generative AI and AI-assisted technologies in the manuscript preparation process

AI-based tools were used to critique drafts of this perspective and to identify opportunities to better communicate with a broader audience.
